# Printed educational messages aimed at family practitioners fail to increase retinal screening among their patients with diabetes: a pragmatic cluster randomized controlled trial [ISRCTN72772651]

**DOI:** 10.1186/1748-5908-9-87

**Published:** 2014-08-06

**Authors:** Merrick Zwarenstein, Susan K Shiller, Ruth Croxford, Jeremy M Grimshaw, Diane Kelsall, J Michael Paterson, Andreas Laupacis, Peter C Austin, Karen Tu, Lingsong Yun, Janet E Hux

**Affiliations:** Centre for Studies in Family Medicine, Department of Family Medicine, Schulich School of Medicine and Dentistry, Western University, 1465 Richmond Street, London, Ontario N6A 3K7 Canada; Institute for Clinical Evaluative Sciences, 2075 Bayview Avenue, Toronto, Ontario M4N 3M5 Canada; Faculty of Medicine, University of Toronto, 1 King's College Circle, Medical Sciences Building, Toronto, Ontario M5S 1A8 Canada; Canadian Medical Association Journal, 1867 Alta Vista Drive, Ottawa, ON K1G 5W8 Canada; Ottawa Hospital Research Institute, The Ottawa Hospital - General Campus, Box 711, Ottawa, Ontario 501 Smyth Road, K1H 8L6 Canada; Faculty of Medicine, University of Ottawa, 451 Smyth Rd, Ottawa, Ontario K1H 8M5 Canada; Canadian Diabetes Association, Toronto, Ontario Canada; Institute of Health Policy, Management and Evaluation, University of Toronto, Health Sciences Building, 155 College Street, Suite 425, Toronto, ON M5T 3M6 Canada; Keenan Research Centre of the Li Ka Shing Knowledge Institute at St Michaels Hospital, 30 Bond Street, Toronto, Ontario M5B 1W8 Canada

## Abstract

**Background:**

Evidence of the effectiveness of printed educational messages in narrowing the gap between guideline recommendations and practice is contradictory. Failure to screen for retinopathy exposes primary care patients with diabetes to risk of eye complications. Screening is initiated by referral from family practitioners but adherence to guidelines is suboptimal. We aimed to evaluate the ability of printed educational messages aimed at family doctors to increase retinal screening of primary care patients with diabetes.

**Methods:**

Design: Pragmatic 2×3 factorial cluster trial randomized by physician practice, involving 5,048 general practitioners (with 179,833 patients with diabetes). Setting: Ontario family practitioners. Interventions: Reminders (that retinal screening helps prevent diabetes-related vision loss and is covered by provincial health insurance for patients with diabetes) with prompts to encourage screening were mailed to each physician in conjunction with a widely-read professional newsletter. Alternative printed materials formats were an ‘outsert’ (short, directive message stapled to the outside of the newsletter), and/or a two-page, evidence-based article (‘insert’) and a pre-printed sticky note reminder for patients. Main outcome measure: A successful outcome was an eye examination (which includes retinal screening) provided to a patient with diabetes, not screened in the previous 12 months, within 90 days after visiting a family practitioner. Analysis accounted for clustering of doctors within practice groups.

**Results:**

No intervention effect was detected (eye exam rates were 31.6% for patients of control physicians, 31.3% for the insert, 32.8% for the outsert, 32.3% for those who received both, and 31.2% for those who received both plus the patient reminder with the largest 95% confidence interval around any effect extending from −1.3% to 1.1%).

**Conclusions:**

This large trial conclusively failed to demonstrate any impact of printed educational messages on screening uptake. Despite their low cost, printed educational messages should not be routinely used in attempting to close evidence-practice gaps relating to diabetic retinopathy screening.

**Trial registration:**

ISRCTN72772651

**Electronic supplementary material:**

The online version of this article (doi:10.1186/1748-5908-9-87) contains supplementary material, which is available to authorized users.

## Background

Gaps between evidence-based ideal clinical practice and what doctors actually do can be responsible for substantial harm [1, 2]. For example, beta-blockers, angiotensin-converting enzyme inhibitors, and lipid-lowering drugs have been shown to reduce re-hospitalizations and mortality following an acute myocardial infarction (AMI), yet these drugs are underprescribed [3]. Printed educational messages (PEMs) directed to doctors may be one way to address such gaps. PEMs have the considerable advantage of low cost and easy dissemination, so even small improvements may be efficiently obtained [4]. However, the evidence on their effectiveness is contradictory. Freemantle *et al.* conducted a systematic review of the effects of PEMs [5]. None of the small number of included trials found statistically significant improvements in practice, leading to the conclusion that PEMs were ineffective. When Farmer *et al.* updated this review [6] using a superior analytic approach (summarising observed effect sizes instead of vote counting studies with statistically significant effects), they observed a median absolute improvement of care of 4.9% (range −8.0% to +9.6%), on par with other much more expensive interventions like audit and feedback, or academic outreach [7]. A further updated review by Giguère *et al.*[8] observed smaller improvements in care (median absolute improvement of care of 2.0%, range 0 to +11.0%), and argued for the need for additional primary research and further investigation of different characteristics of PEMS in head-to-head comparisons. These contradictory findings leave policy makers uncertain about the role of PEMs, at a time when closing evidence-to-practice gaps may offer a more cost-effective investment of health system resources than developing new interventions [9].

Reviews cannot overcome limitations of the primary evidence. There are few trials of PEMs, often small in size with methodological weaknesses (for example unit of analysis errors in cluster randomized trials). To clarify the usefulness of PEMs, large, pragmatic [7, 10], and well-designed randomized controlled trials on the effect of PEMs on guideline adherence, conducted in real world settings, amongst typical practitioners are needed. The Ontario Printed Education Materials (OPEM) trial on retinopathy screening was such a trial: a large pragmatic cluster randomised trial powered to be able to detect small effects.

In Ontario, diabetes mellitus (diabetes) affects over 15% of women and 19% of men aged 65 years and older. Diabetic retinopathy is a common complication, affecting about 70% of persons with type 1 diabetes and 40% of persons with type 2 diabetes, and is the leading cause of preventable blindness in Canadians aged 30 to 69 [11]. Most vision loss related to diabetic retinopathy can be prevented through retinal screening examinations and appropriate treatment. Early treatment has been shown to decrease the risk of severe vision loss from proliferative diabetic retinopathy by 90% and the risk of vision loss from macular oedema by 50% [12]. Evidence-based practice guidelines in Canada call for screening for retinopathy at the time of diagnosis and every two years thereafter for everyone with type 2 diabetes [13].

Under the Ontario Health Insurance Plan (OHIP) Ontario residents have universal public health insurance, including free at point of service access to family practitioner (FP) care. Eye examinations (which include retinal screening), performed by an optometrist, FP, or ophthalmologist, are also an insured service, and thus are free at point of service to patients without any out-of-pocket payment, for all adults aged 65 years and older and also for those under age 65 who have been diagnosed with diabetes.

Yet in Ontario, the observed screening rates within the first year of diabetes diagnosis falls far below recommended levels (43% and 50% for diabetes patients aged 30 to 49 and 50 to 64 respectively).

If the impact on screening uptake of PEMs was indeed 5% improvement, then at the trivial cost of a letter to each FP, Ontario’s rate of screening and treatment, especially among younger patients, could be substantially increased, averting blindness in more than 100 Ontarians per annum [14].

We report here a trial of PEMs, evaluating their impact on screening rates for diabetic retinopathy. The objective of the study was to determine whether sending a PEM to FPs (and the format of the PEM) would affect the likelihood that a patient visiting their doctor would receive an eye exam within 90 days of their visit.

## Methods

These are presented in detail in our published protocol [15].

### Study design and randomisation

The study was a pragmatic, factorial, cluster-randomized controlled trial (Table 1). The study intervention was mailed on 1 April 2005 and the follow up period extended from 1 July 2005 to 30 June 2006. To prevent contamination (sharing of information among doctors in group practice) we randomised at the level of the practice. FPs were placed into practices on the basis of a shared street address. Practices were randomly assigned to an intervention group by the study statistician, using computer-generated random numbers.

**Table 1:** **Study design and number of practice groups**/**number of physicians**

	Randomized		Included in the analysis (saw at least one patient with diabetes during the follow-up year)	
Intervention	Number of practice groups	Number of physicians	Number of practice groups	Number of physicians
1. *informed* only (no PEM*)	1,077	1,318	1,051	1,282
2. *informed* plus insert	1,066	1,305	1,042	1,273
3. *informed* plus outsert				
a. No patient reminder notepad	535	642	519	623
b. Patient reminder notepad	536	643	523	629
4. *informed* plus insert & outsert				
No patient reminder notepad	535	632	527	620
Patient reminder notepad	533	639	519	621
Total	4,282	5,179	4,181	5,048

*PEM: Printed Educational Message.

### Study population

The study participants were all FPs with an active practice in Ontario in 2003/2004. ‘Active’ practice was defined as a total billing volume for the year of at least $50,000 and writing prescriptions for at least 100 different patients, with at least one prescription in at least 10 of the 12 months.

The Ontario Diabetes Database was used to identify Ontario residents diagnosed with diabetes on or before 31 March 2005. Individuals were included if they were at least 30 years old on 31 March 2005, were alive at the end of the follow-up period (30 June 2006), and visited one of the target FPs within one year of the intervention mail-out.

Patients who had an eye exam in the preceding nine months were excluded from the study.

### The printed educational materials interventions

*Informed* was a free, peer-reviewed, evidence-based primary care practice synopsis, written and produced by clinical and research staff from the Institute for Clinical Evaluative Sciences (ICES, http://www.ices.on.ca), and mailed to nearly 15,000 health care providers in Ontario from 1994 to 2007.

We designed two types of PEM to address the identified evidence-practice gap: a short, directive, evidence-based PEM on a postcard-sized card stapled to the front page of *informed* (the ‘outsert’); and a two-page insert, indistinguishable from the rest of *informed* in size and style (the ‘insert’). The insert excluded the directive statements of the outsert but included more background, a summarised evidence-based guideline, and references. Both were developed over three months of meetings with input from five ICES staff with knowledge translation experience (an internist, two family physicians, and two knowledge translation researchers), the writing team of the *informed* newsletter and a communications consultant.

We also issued a pad of take-home reminders (aimed at patients, to remind them to make an appointment for an eye exam), to be given to patients by their FP. Because it was not clear whether the reminder would be any more effective than the doctor’s verbal advice, doctors randomised to receive the outsert were also randomized to receive or not receive a pad of patient-aimed reminder slips.

Insert, outsert, and patient reminder were included with the 1 April 2005 edition of *informed*.

Practices were randomly assigned to one of four intervention groups. The two intervention groups selected to receive an outsert were further randomly divided into two subgroups, one of which received the patient reminder notepad.

**Figure 1:**
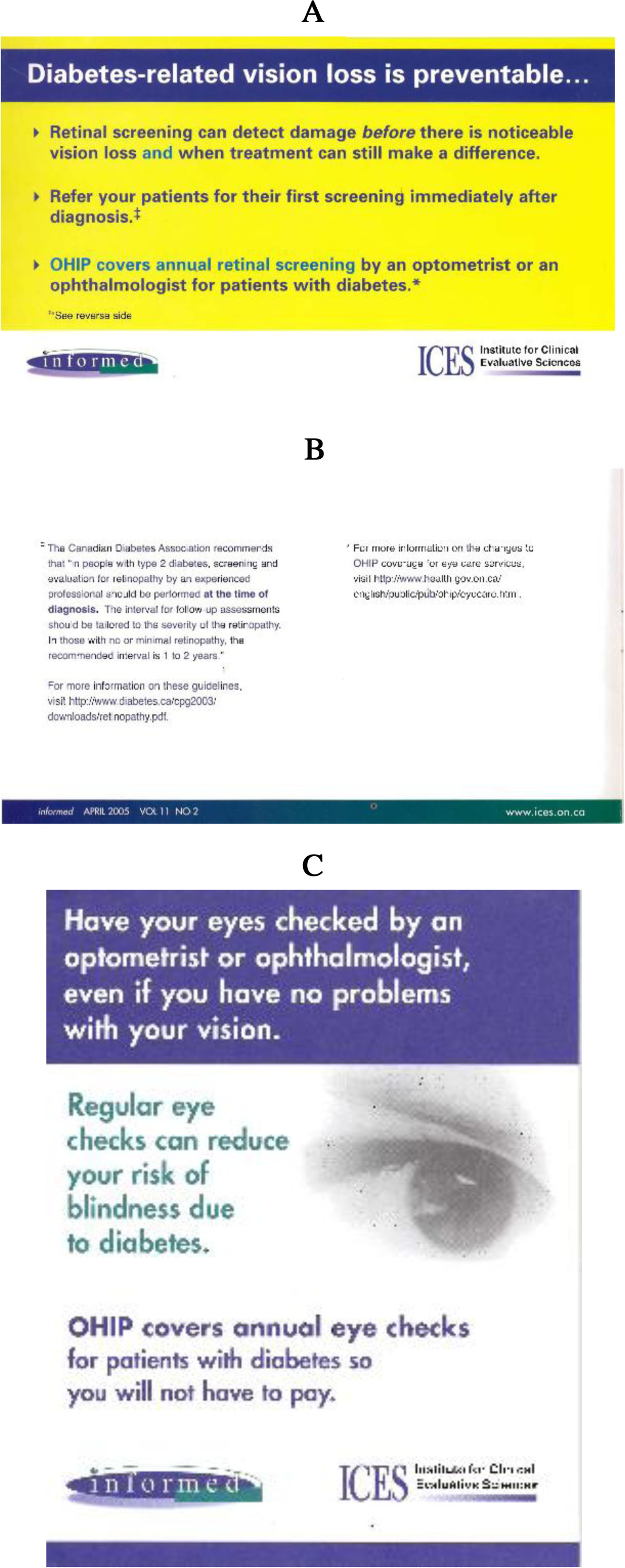
**Interventions:** **the outsert and patient memo.**

Figure 1 shows the outsert and patient reminder. The full insert is included as Additional file 1.

### Data sources

The following administrative data sources were used [16]. These data sets were held securely in a linked, de-identified form and analyzed at the Institute for Clinical Evaluative Sciences.

1. The OHIP Claim History Database details payments to health care professionals, including an encrypted provider number unique to each health care professional, an anonymous, encrypted patient identifier unique to each patient, the service provided, and the service date.
2. The Registered Persons Database contains basic demographic information for each insured person.
3. The Corporate Provider Database contains limited demographic and practice information for each practitioner.

Finally, we used the Ontario Diabetes Database, a validated registry of Ontario residents diagnosed with diabetes [17].

Records from these databases were linked, using the patient and provider identifiers, to determine which individuals with diabetes visited one of the target physicians in the year following the intervention, and whether that individual subsequently received an eye examination.

### Outcomes

The primary outcome measure was whether or not an eligible trial patient received an eye exam within 90 days of their first FP visit during the one-year period following the mail-out. Because the PEMs specifically addressed the issue of insured eye exams for patients with diabetes younger than 65 years, a secondary analysis examined the impact of patient age on the uptake of eye exams.

### Power

Based on Monte Carlo simulations, assuming an intra-cluster correlation coefficient of 0.1 [18], three patients with diabetes per physician, and a baseline rate of screening of 36%, a trial with 1,250 practices per arm would provide over 97% power to detect an absolute increase of 5% in the screening rate, and over 98% power to distinguish between the effects of the combined intervention and either alone, assuming the effect of each to be 5% and the combined effect to be additive.

### Statistical analysis

Analysis was on an intention-to-treat basis. Logistic regression models, estimated using generalized estimating equations (GEE) methods to account for the clustering of patients within physician practices, were used to estimate the effect of the intervention on the likelihood of patient screening. Two logistic regression models were fitted: one including only the interventions (insert and/or outsert and the patient reminders), and a second also adjusting for patient- and physician-level covariates. All analyses were performed using SAS version 9.1 (SAS Institute, Cary, North Carolina). Two-tailed p-values less than or equal to 0.05 were considered to be significant.

### Ethics

This study was approved by the Research Ethics Board at Sunnybrook Health Sciences Centre.

## Results

### Physician and patient selection

**Figure 2:**
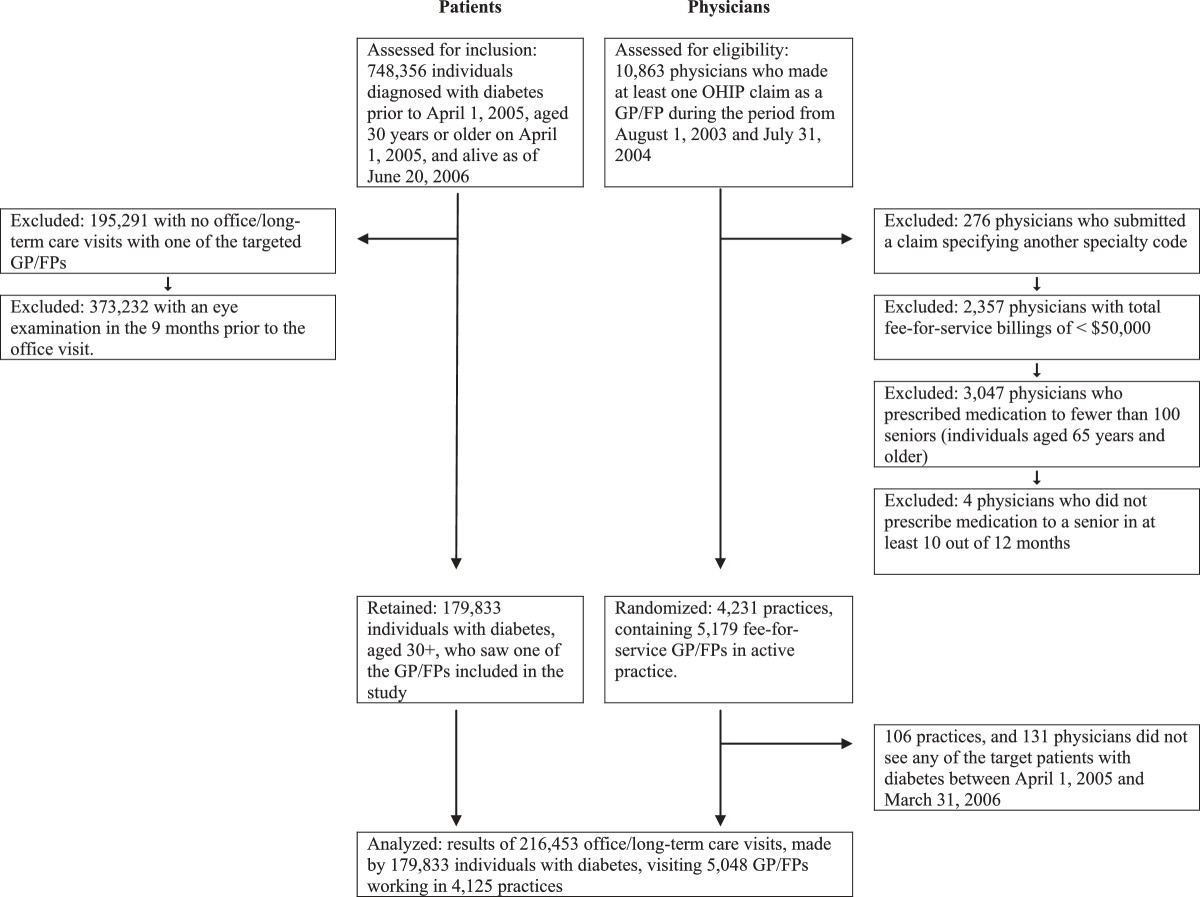
**Patient and physician selection:** **consort diagram.**

Figure 2 shows the number of physicians and patients included in the study. Three-quarters of patients with diabetes who met the inclusion criteria visited one of the physicians targeted by this study during the follow up period. However, two-thirds of these individuals had already had an eye examination in the nine months immediately prior to the office visit and were excluded (Figure 2).

### Baseline characteristics

There were small, clinically unimportant, differences between the demographics of patients with diabetes who paid a visit to a study physician and those who did not, and between those who were and were not included in the analysis (Table 2). Patients aged 65 years and older were significantly more likely to visit a primary care doctor, and also more likely to have received a recent eye exam (and hence to be excluded from the analysis).

**Table 2:** **Characteristics of individuals with diabetes**

		Individuals with diabetes who had at least one office visit to a general/family physician in 2005/06	
Patient characteristics as of April 1, 2005	Excluded from the analysis due to no office visit	Excluded from the analysis due to a recent eye exam^†^	Patients included in the analysis
	(N = 193,674)	(N = 373,232)	(N = 179,833)
Sex (% male)	52.9	51.4	51.2
% aged ≥66* years	39.6	47.9	42.5
Mean age in years (SD)	60.3 (14.6)	62.9 (13.9)	61.7 (13.1)
Total number of visits to a general/family physician made by the patient during 2005/06: median (25th, 75th percentiles)	None	6 (3, 9)	6 (3, 10)
Years with diabetes: mean (SD)	7.1 (4.5)	6.9 (4.5)	6.7 (4.4)

^†^An eye examination within 9 months prior to the office visit, making the individual ineligible for an insured complete eye examination within the next 90 days.

There were no meaningful physician differences among the intervention groups (Table 3).

**Table 3:** **Physician characteristics, by intervention group**

	Intervention group with number of physicians							
	Informed only	Insert	Outsert	Outsert + Patient Reminder	Insert + Outsert	Insert + Outsert + Patient reminder	All	P-value^‡^
	N = 1282	N = 1273	N = 623	N = 629	N = 620	N = 621	N = 5048	
Number of patient visits	55,961	53,748	26,379	27,392	26,061	26,912	216,453	
Gender (% male)	77.2	77.5	74.8	77.0	77.6	74.4	76.6	0.55
Place of training (%)								0.77
Canada	76.4	76.8	76.6	77.0	78.1	77.9	77.0	
Solo practice (%)	68.4	68.7	68.1	70.3	73.4	69.9	69.5	0.27
Rural* (%)	11.9	12.3	12.5	12.1	11.8	12.9	12.2	0.99
Years since graduation: mean (std)	26.9 (10.5)	26.5 (10.2)	26.0 (10.5)	26.3 (10.3)	26.8 (10.1)	26.4 (9.9)	26.5 (10.3)	0.62

^‡^P-value testing the null hypothesis that there was no difference among the intervention groups. The proportion of patients receiving an eye exam was compared using GEE.

*A practice area was designated as rural if it was located in a geographic region with a population smaller than 10,000.

### Analysis of intervention effects

Intervention effects are shown in Table 4. Neither the unadjusted nor the adjusted results show any evidence that the interventions (alone or in combination), were effective in increasing rates of eye examination among eligible trial patients. The widest confidence interval reported in the table, an odds ratio between 0.94 and 1.04 for the insert + outsert + reminder notepad, corresponds to a true absolute effect of the intervention lying between a decrease in retinal screening of 1.3% and an increase of 1.1%. Thus, the estimate of effect for the presumably strongest ‘dose’ of intervention is of trivial clinical value, is not statistically significantly superior compared no intervention, and the confidence interval does not contain values of clinical importance.

**Table 4:** **Results**

Percentage of patients obtaining retinal screening within 90 days of mail out (Crude success rate)*			
Intervention	Median success rate (percent)	25th and 75th percentile success rates (percent)	P-value
Informed only (reference group)	31.0	25.0, 37.0	0.96
+ insert	30.9	25.3, 37.8	
+ outsert, no reminder notepad	30.8	25.0, 37.1	
+ outsert and reminder notepad	30.4	25.0, 37.5	
+ insert and outsert, no reminder notepad	30.3	25.0, 37.3	
+ insert and outsert and reminder notepad	30.4	25.0, 37.5	
Overall	30.8	25.0, 37.5	
**Regression Model: Unadjusted**			
**Intervention**	**Odds ratio**	**95% confidence interval**	**P-value**
Informed only (reference group)	1.00		0.97
+ insert	1.00	0.96 to 1.03	
+ outsert, no reminder notepad	0.99	0.95 to 1.05	
+ outsert and reminder notepad	0.98	0.93 to 1.03	
+ insert and outsert, no reminder notepad	0.99	0.94 to 1.04	
+ insert and outsert and reminder notepad	0.99	0.94 to 1.04	
**Regression Model: Adjusted for patient and physician covariates** ^**†**^			
**Intervention**	**Odds ratio**	**95% confidence interval**	**P-value**
Informed only (reference group)	1.00		0.66
+ insert	0.99	0.95 to 1.03	
+ outsert, no reminder notepad	0.96	0.91 to 1.01	
+ outsert and reminder notepad	0.96	0.91 to 1.02	
+ insert and outsert, no reminder notepad	0.98	0.93 to 1.04	
+ insert and outsert and reminder notepad	0.97	0.92 to 1.02	

*In order to present the quartiles, the percentage of patients receiving an eye examination was determined for each physician, and these percentages were summarized for each intervention group. Group practices were not taken into account for this crude analysis.

^†^The model was adjusted for these patient variables: age, gender, length of time diagnosed with diabetes, and whether the patient had an eye examination at any time in the two years prior to the office visit. The model was adjusted for these physician variables: year of graduation, gender, place of training, type of practice (solo/group), place of practice (rural/urban), and elapsed time between the mail-out and the office visit.

While the probability of having an eye examination depended on patient age and rises sharply at age 65, there was no indication that intervention effectiveness varied with patient age.

The intracluster correlation coefficient was 0.024 (95% confidence interval 0.022 to 0.025).

## Discussion

In the face of the previous uncertainty about the effectiveness of printed educational materials in changing physician behaviour, this study, with sufficient power to detect even a small effect, failed to change practitioner behaviour, as measured by retinal screening rates among patients with diabetes. The intervention was a faithful operationalisation of the sort of printed educational materials that are routinely used and explored both long and short formats; this can be regarded as an evaluation of a realistic intervention.

The effectiveness of printed educational materials depends on the message being received by the physicians at whom the PEMs were aimed, read, judged credible, transmitted to (and acted on by) patients who might benefit from screening. The first three steps appear likely to have occurred: The reliability of the postal services in Canada are such that we can be confident that the materials were successfully delivered. They are also likely to have been read and judged credible: In 1997, The Strategic Council Inc., a market research organisation, contacted 500 Ontario physicians by phone to determine readership and recall of *informed*. They found that 71% of the respondents recalled receiving *informed* and that of these, 89% found it useful or very useful and 53% read most or every issue (internal report, personal communication, Dianne Kelsall, editor of *informed*).

It is possible that the physicians did recommend screening but did not make a referral or that patients did not keep their ophthalmology appointments. However, given that eye examinations are free for Ontario patients with diabetes and for all people aged 65 and over (in which group eye examination among patients with diabetes rises to 89% coverage within one year of diagnosis), it seems unlikely that patients with diabetes would fail to take up their physician’s recommendation simply because they are not yet 65. Thus patient failure to respond to the physician recommendation seems to be an unlikely explanation for the lack of impact of our intervention. We believe that the message was likely received, read, and judged credible by physicians, and would not have been ignored by patients. Yet PEMs were not successful at improving patient screening rates.

This suggests that PEMs simply failed to improve physician knowledge regarding the availability of free tests for patients with diabetes who were aged under 65; alternatively the PEMs failed to either influence the transmission of this message to patients or to lead to referral.

The content of our PEMs was not based on a prior assessment of barriers to screening and so it is possible that these are not the relevant barriers. Perhaps the actual barriers to improved screening lie elsewhere, with possibilities including the organisation of screening or patient factors such as motivation or geographical access. These potential barriers are indicated by the process study conducted in parallel with this RCT on a subsample of practitioners, and reported alongside [19].

This study is reliable and was conducted under realistic conditions to mimic typical programme delivery. The use of administrative data as the source of our outcome measure allowed us to include nearly the complete population of family practitioners and all of their eligible patients, to be able to provide a definitive result. This trial is one of the largest published randomised trials of knowledge translation or implementation to date, and given its power a false negative result is unlikely.

## Conclusions

This real-world study supports the conclusion that PEMs, whether long and discursive or short and directive, and with or without patient reminder notes, do not change physician behaviour as measured by adherence to evidence based screening recommendations for diabetic retinopathy. These results strongly suggest that the use of PEMs alone is not a useful strategy for closing this evidence-practice gap, and underline the importance of continuing to explore complementary or alternative behaviour change interventions. We have replicated these results for two prescribing behaviours (in preparation) and further replication for diagnostic and other therapeutic behaviours would be informative.

## Authors’ information

Retired: Pamela M. Slaughter.

## Electronic supplementary material

Additional file 1: Graphics file.(PDF 494 KB)

Below are the links to the authors’ original submitted files for images.

Authors’ original file for figure 1

Authors’ original file for figure 2
