# Supplementary material for: Printed educational messages aimed at family practitioners fail to increase retinal screening among their patients with diabetes: a pragmatic cluster randomized controlled trial [ISRCTN72772651]
Source: Implement Sci. 2014 Aug 6;9:87. doi: 10.1186/1748-5908-9-87 (PMC4261896; doi:10.1186/1748-5908-9-87)
Supplement: Supplementary file 1 — Additional file 1: Graphics file.(PDF 494 KB) [file 13012_2013_826_MOESM1_ESM.pdf]

informed Xtra

APRIL 2005 VOL 11 NO 2

Eye Spy

Retinopathy screening in persons with diabetes

About 70% of patients with type 1 diabetes mellitus (DM) and 40% of those with type 2 DM develop a common complication of the disease—diabetic retinopathy, which is a serious threat to vision and the leading cause of blindness in Canadians aged 30 to 69 years. Many of those with vision-threatening disease may not have symptoms. Most vision loss resulting from diabetic retinopathy (DR) can be prevented through periodic retinal screening and laser photocoagulation treatment when needed.

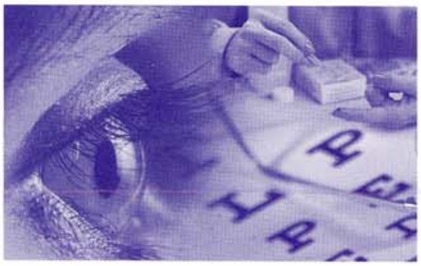

The Canadian Diabetes Association (CDA) guidelines recommend that screening be initiated *five* years after diagnosis of type 1 diabetes in all individuals aged 15 years or older. Persons newly diagnosed with type 2 DM should undergo retinal screening *at* diagnosis. In 2003, an ICES analysis of eye examinations conducted in persons with DM in Ontario revealed that screening rates for diabetic retinopathy fall well below the latter recommendation. In fact, only 51% of persons with type 2 DM had an eye examination within *one year* of diagnosis.

Why screen?

Diabetic retinopathy is due to microangiopathy affecting the retinal blood vessels. Both microvascular leakage (from breakdown of the inner retinal-blood barrier) and microvascular occlusion cause the characteristic damage. Early non-proliferative "background" diabetic retinopathy is found in almost all persons with type 1 DM and 60% of those with type 2 DM within 20 years of diagnosis. This stage is characterized by the development of microaneurysms, hemorrhages, hard exudates (lipid deposits) and retinal edema. While non-proliferative retinopathy usually does not lead to visual impairment, leakage of fluid in the area of the macula (macular edema) is an important cause of blindness in patients with diabetes.

Non-proliferative retinopathy can progress to the proliferative form, which is typified by the development of new retinal blood vessels that have a tendency to bleed, leading to other potentially blinding complications. Fewer than 10% of people with type 2 DM will develop proliferative diabetic retinopathy within 20 years of initial diagnosis, while half of persons with type 1 DM will develop the disease within the same time frame.

With early detection, vision loss due to diabetic retinopathy can be prevented by laser photocoagulation of the retina. If left untreated, up to 50% of patients with proliferative retinopathy will be blinded within five years. Randomized trials have convincingly demonstrated that early laser treatment (retinal photocoagulation) of sight-threatening diabetic retinopathy decreases the risk of severe vision loss from proliferative DR by 90% and the risk of vision loss from macular edema by 50%. One important finding from these studies was that the effectiveness of treatment is optimal *before* vision loss occurs and falls sharply if applied later.

Vision loss in DM can also result from an increased occurrence of cataracts and open angle glaucoma.

When to start screening

Screening approaches for diabetic retinopathy differ in type 1 and type 2 DM based on the natural history of the disease. Diabetic retinopathy rarely develops in children under the age of ten years with type 1 DM, regardless of how long the child has had the disease. However, the prevalence rises sharply after five years of diabetes in postpubertal individuals with type 1 DM. Among children under 15 years of age, the prevalence of mild non-proliferative retinopathy is about 2%; no studies report proliferative retinopathy in this age group. Thus, the recommendation is that screening should begin *five years* after diagnosis of type 1 diabetes in all individuals aged 15 years or older.

In older persons with type 2 DM, diabetic retinopathy may already be present when the diagnosis of DM is made, due to delayed detection of the diabetes. Between 21–39% of patients with type 2 DM may have retinopathy at or soon after the time of diagnosis. Thus, the guidelines recommend that persons newly diagnosed with type 2 DM undergo retinal screening *at* diagnosis.

How to screen

Approaches to screening for diabetic retinopathy include direct ophthalmoscopy, indirect slit-lamp fundoscopy and retinal photography, used alone or in combination, with subsequent grading of the degree of retinopathy. Photography provides a permanent and comparative record for monitoring progressive change. If screening cannot be done locally, a retinal photograph may be taken by a trained professional and digitally transmitted to an eye care professional for reading.

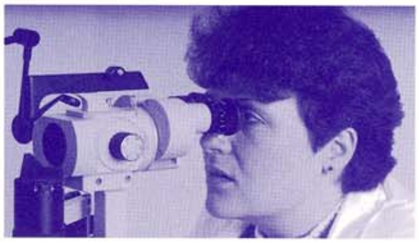

There is widespread agreement that screening for diabetic retinopathy should involve a *dilated* examination of the retina by a highly trained examiner. Examinations through undilated pupils miss about 50% of patients with retinopathy. Due to the equipment required, screening generally necessitates referral to an ophthalmologist or optometrist.

Continued ▸ 2

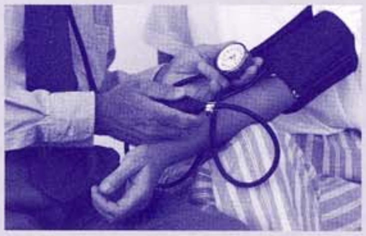

Preventing diabetic retinopathy

The development and progression of retinopathy may be prevented through intensive diabetes management:

- ✔ Achieving optimal metabolic control.
- ✔ Treatment of elevated blood pressure and lipid levels.

Adapted from: *CMAJ* 1998; 159(Suppl 8):S1–S29.

How often to screen

The interval for follow-up assessments should be tailored to the absence presence and severity of retinopathy (see table below). If retinopathy is not present, the CDA guidelines recommend:

- ✔ Annual screening for those with type 1 DM.
- ✔ Screening every one to two years for those with type 2 DM.

If retinopathy is present, an appropriate monitoring interval (one year or less) should be established. Proliferative or severe non-proliferative retinopathy necessitates referral to an ophthalmologist or retinal specialist with access to surgical facilities.

The Bottom Line

- **Patients with vision-threatening diabetic retinopathy may have no symptoms.**
- **Timely laser photocoagulation therapy can help prevent vision loss.**
- **Persons newly diagnosed with type 2 DM should undergo retinal screening at diagnosis.**
- **Due to the equipment required, screening for diabetic retinopathy necessitates referral to an ophthalmologist or optometrist.**

Peer-reviewed Article

When to initiate screening	Screening methods	If retinopathy is present	If retinopathy is not present
✔ Five years after diagnosis of type 1 diabetes in all individuals ≥15 years of age.	✔ 7-standard field, stereoscopic-colour fundus photography with interpretation by a trained reader (gold standard).	✔ Diagnose retinopathy, severity and establish appropriate monitoring intervals (1 year or less).	✔ Type 1 diabetes: rescreen annually.
✔ In all individuals at diagnosis of type 2 diabetes.	✔ Direct ophthalmoscopy or indirect slit-lamp fundoscopy through dilated pupil.	✔ Treat sight-threatening retinopathy with laser therapy.	✔ Type 2 diabetes: rescreen every 1–2 years.
	✔ Digital fundus photography.	✔ Review glycemic, BP and lipid control and adjust therapy to reach targets as per guidelines.	✔ Review glycemic, BP and lipid control and adjust therapy to reach targets as per guidelines.
		✔ Screen for other diabetes complications.	✔ Screen for other diabetes complications.

Adapted from: Canadian Diabetes Association Guidelines. http://www.diabetes.ca/cpg2003/chapters/table1screeningforretinopathy.htm (accessed 31 Jan 2005).

For more information, see the Canadian Diabetes Association 2003 clinical practice guidelines available at http://www.diabetes.ca/cpg2003/ and the *"Diabetes in Ontario: An ICES Practice Atlas"* at http://www.ices.on.ca, under the Publications section.

informed
Institute for Clinical Evaluative Sciences (ICES), G1 06, 2075 Bayview Avenue, Toronto, ON M4N 3M5
Phone: (416) 480-4055, Extension 5168 Internet: http://www.ices.on.ca E-mail: informed@ices.on.ca
