# Supplementary figures and images for: Printed educational messages aimed at family practitioners fail to increase retinal screening among their patients with diabetes: a pragmatic cluster randomized controlled trial [ISRCTN72772651]
Source: Implement Sci. 2014 Aug 6;9:87. doi: 10.1186/1748-5908-9-87 (PMC4261896; doi:10.1186/1748-5908-9-87)

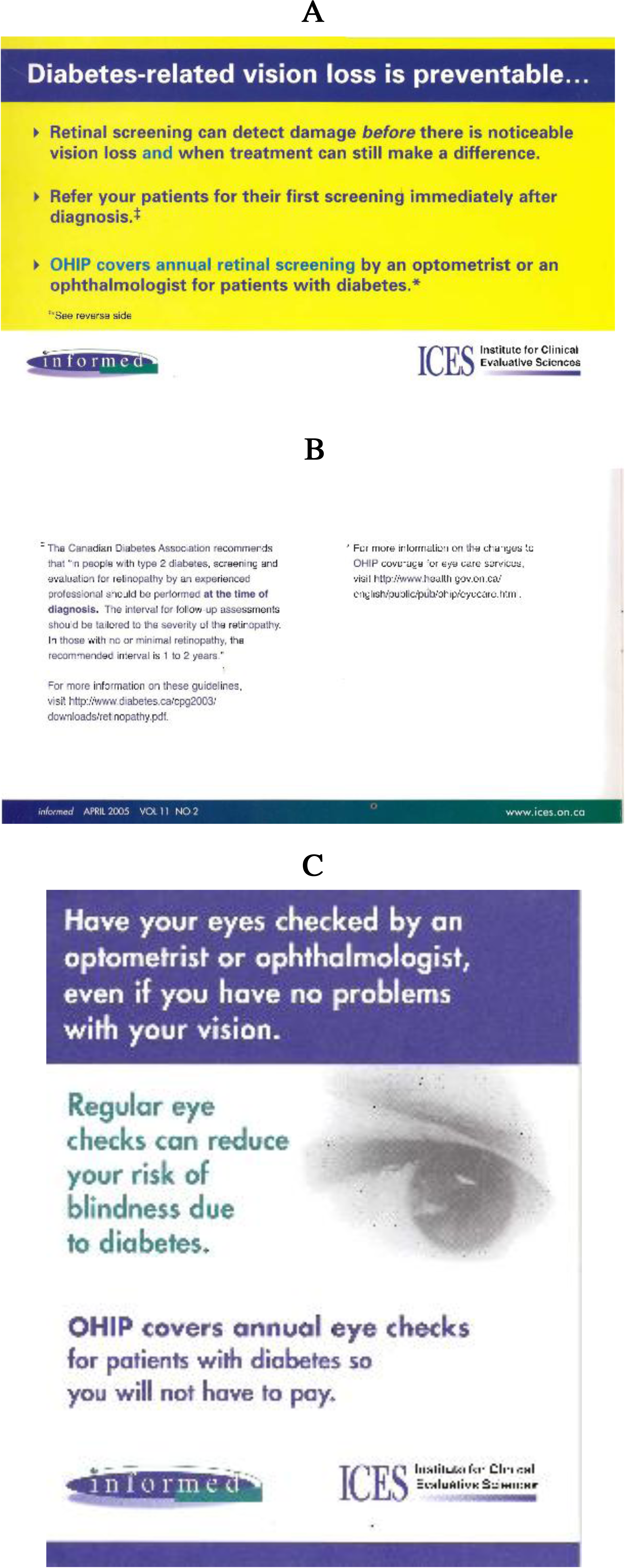

Supplement: Supplementary file 2 — Authors’ original file for figure 1 [file 13012_2013_826_MOESM2_ESM.tif]

## Patients

## Physicians

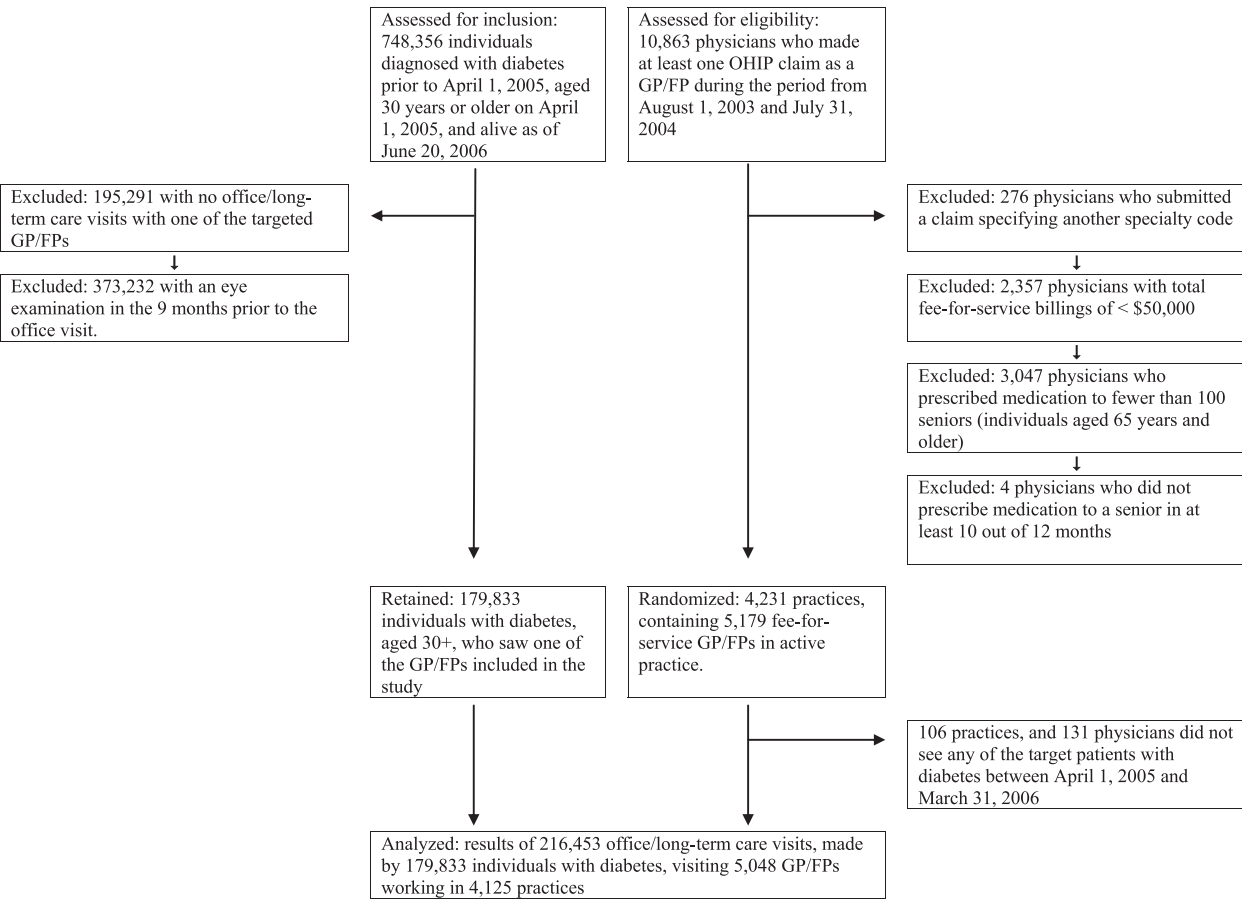

Supplement: Supplementary file 3 — Authors’ original file for figure 2 [file 13012_2013_826_MOESM3_ESM.pdf]
